# Brain Immune Interactions—Novel Emerging Options to Treat Acute Ischemic Brain Injury

**DOI:** 10.3390/cells10092429

**Published:** 2021-09-15

**Authors:** Sajjad Muhammad, Shafqat Rasul Chaudhry, Ulf Dietrich Kahlert, Mika Niemelä, Daniel Hänggi

**Affiliations:** 1 Department of Neurosurgery, Faculty of Medicine and University Hospital Düsseldorf, Heinrich-Heine University Düsseldorf, Moorenstrasse 5, D-40225 Düsseldorf, Germany; ulf.kahlert@med.uni-duesseldorf.de (U.D.K.); daniel.haenggi@med.uni-duesseldorf.de (D.H.); 2 Department of Neurosurgery, University of Helsinki and Helsinki University Hospital, 00100 Helsinki, Finland; mika.niemela@hus.fi; 3 Department of Neurosurgery, University Hospital Bonn, D-53127 Bonn, Germany; shafqatrasul@yahoo.com; 4 College of Pharmaceutical Sciences, Shifa Tameer-e-Millat University, Islamabad 44000, Pakistan

**Keywords:** ischemic stroke, inflammation, immune cells, neuroimmune axis, stem cell

## Abstract

Ischemic stroke is still among the leading causes of mortality and morbidity worldwide. Despite intensive advancements in medical sciences, the clinical options to treat ischemic stroke are limited to thrombectomy and thrombolysis using tissue plasminogen activator within a narrow time window after stroke. Current state of the art knowledge reveals the critical role of local and systemic inflammation after stroke that can be triggered by interactions taking place at the brain and immune system interface. Here, we discuss different cellular and molecular mechanisms through which brain–immune interactions can take place. Moreover, we discuss the evidence how the brain influence immune system through the release of brain derived antigens, damage-associated molecular patterns (DAMPs), cytokines, chemokines, upregulated adhesion molecules, through infiltration, activation and polarization of immune cells in the CNS. Furthermore, the emerging concept of stemness-induced cellular immunity in the context of neurodevelopment and brain disease, focusing on ischemic implications, is discussed. Finally, we discuss current evidence on brain–immune system interaction through the autonomic nervous system after ischemic stroke. All of these mechanisms represent potential pharmacological targets and promising future research directions for clinically relevant discoveries.

## 1. Introduction

### 1.1. Epidemiology and Pathophysiology of Stroke

Stroke is the third leading cause of death following heart diseases and cancer. Stroke affects around 15 million people yearly worldwide [1]. The majority of the strokes are ischemic [2], but still approximately two million cases are the hemorrhagic type of stroke [3]. Moreover, stroke is the leading cause of long-term disability worldwide and poses a profound economic burden [1]. Despite years of intensive research, therapeutic options are still limited to recanalization strategies either by thrombectomy utilizing stent retrievers [4,5] or thrombolysis with recombinant tissue plasminogen activator (rtPA) with a limited time window up to 4.5 h [6]. Both therapeutic options have their limitations and can lead to life threatening intracranial bleeding. All efforts to search for an effective neuroprotective drug for cerebral ischemia have proved futile. Due to limitations of therapeutic options in clinical practice, there is an urgent need to explore and identify new treatment targets located within and outside the brain.

### 1.2. Inflammation and Immune Response after Cerebral Ischemia

Ample evidence suggests that local and systemic inflammation plays a pivotal role in cerebral ischemia [2,7,8,9,10,11,12,13,14,15]. The pathophysiology of cerebral ischemia is, however, complex. Reduction of blood supply to the brain initiates the ischemic cascade that leads to neuronal death and rapid loss of neuronal function [2]. This ischemic cascade is characterized by the activation of several cellular signaling pathways that compromise cell survival and function [16]. Furthermore, ischemic stroke triggers blood–brain barrier (BBB) disruption, thus contributing to the secondary progression of ischemic injury by increasing brain edema and exacerbating the inflammatory response in the subacute phase [2,17]. The severity of these early events influences the capacity of neurons to recover in the chronic phase.

The injury of nervous tissue leads to release of neuronal antigens, ATP and damage-associated molecular patterns (DAMPs) that elicit activation of immune response. Moreover, the release of chemokines and cytokines released from the injured brain can also activate the immune system and increased expression of adhesion molecules facilitates immune cell infiltration. Furthermore, the autonomic nervous system releases acetylcholine and norepinephrine, which can also regulate the immune system. The dynamics of activation of different immunological players after stroke varies. The innate immune system initiates an early response against brain injury. The cells of innate immunity within the brain (microglia) sense the brain injury through pattern recognition receptors including TLRs and NOD-like receptors (NLRs) [18] leading to activation of downstream pathways such as NF-κB and MAPK pathways resulting in production of proinflammatory cytokines, chemokines and reactive oxygen species (ROS). Similarly, bone-marrow-derived innate immune cells including macrophages, neutrophils, dendritic and natural killer cells infiltrate into the brain through disrupted blood–brain barrier and initiate an acute, nonspecific and quick response that is relatively similar to the intrinsic innate immune response [18]. On the other hand, infiltrated cells of the adaptive immune system, such as T cells, initiate a delayed, but specific response that plays a key role during repair and remodeling [18]. Coordinated crosstalk between the injured brain and peripheral immune system is important to determine the clinical outcome.

## 2. Means of Interaction between Brain and Immune Cells

After cerebral injury, the brain and immune system influence each other through multiple mechanisms and in a very specific way. This bidirectional neuroimmune communication might be important to regulate repair, regeneration and vascular remodeling [19]. How the brain activates the immune system and through which molecules brain–immune interactions take place is an emerging frontier and is under intensive investigations. There is now an ample evidence that both the injured brain and the immune system communicate with each other to reestablish the homeostasis [20]. There are multiple ways to facilitate this communication, including brain-derived antigens, danger-associated molecular patterns (DAMPs), immune signaling molecules and signals via sympathetic and parasympathetic nervous system.

### 2.1. Activation of Immune System through Brain Derived Antigens

A tight blood–brain barrier (BBB) warrants brain to be relatively immune privileged compared to peripheral organs. However, accumulating data have shown that in different pathological conditions, disrupted BBB facilitates a bidirectional communication between brain and immune cells. Brain derived antigens released via disrupted BBB into systemic circulation encounter immune cells and initiate immune response. A prominent example for such a communication is multiple sclerosis, where the immune system is exposed to myelin antigens [21] resulting in infiltration of antigen specific T-cells in the brain that cause demyelination [22]. Disrupted BBB during stroke allows antigens to enter from the brain into the systemic circulation and interact with immune cells. The studies in human stroke exhibit increased titers of antibodies against neurofilaments and N-methyl-D-aspartic acid (NMDA) receptor, showing the previous contact with these antigens [23,24]. Furthermore, microtubule-associated protein 2 (MAP2), NMDA receptor subunit NR-2A, myelin basic protein (MBP) and myelin oligodendrocyte glycoprotein [25] were found in tonsils and cervical lymph nodes of patients with stroke [26]. All these antigens are able to induce autoreactive T cell response. Moreover, stroke patients show consistently elevated activated (CD69+) T cells. All these lines of evidences support the notion that brain-derived antigens are one of the sources of communication between CNS and immune system.

### 2.2. Brain–Immune Interaction through DAMPs

Damage-associated molecular patterns are a set of molecules derived from stressed, injured and dead cells consisting of components such as DNA/RNA and intracellular proteins [27,28]. There is a variety of different DAMPs released from different types of cells and from different tissues upon injury. High-mobility group box-1 (HMGB1), ATP and S100 proteins are the most common DAMPs that critically influence immune response after brain injury. Following is a very brief description of some important DAMPs implicated in mediating immune responses of the injured brain.

#### 2.2.1. Adenosine Triphosphate (ATP)

Cerebral ischemia leads to rapid energy failure, loss of ionic gradient, depolarization and depletion of intracellular ATP and its outflow into the interstitial space [29]. Elevated extracellular ATP acts on purinergic receptors and activate immune cells leading to release of proinflammatory cytokines and activation of inflammasome in neurons and astrocytes [30]. Interestingly, systemic administration of ATP leads to poor outcome after experimental stroke [31] and an antagonist of ATP receptor P2X7 shows protective effect in a model of transient global ischemia via dampening the inflammatory response [32].

#### 2.2.2. S100B

S100B is another DAMP that is mainly expressed in astrocytes in CNS and is released during cerebral ischemia. S100B has been an established surrogate marker for the severity of brain injury and predictive biomarker for the prognosis of stroke [33]. Mice over expressing S100B protein have shown enlarged infarct size after permanent MCA occlusion [34]. S100B can bind to microglial and macrophage receptor RAGE that activates downstream transcription factors including NF-κB and AP-1 as well as the expression of proinflammatory cytokines IL-1β and TNF-α [35].

#### 2.2.3. High-Mobility Group Box-1 (HMGB1)

HMGB1 is another important DAMP that is expressed in all nuclear cells and binds to DNA to regulate gene transcription. During cell death, extracellular release of HMGB1 functions as a potent proinflammatory molecule. HMGB1 can bind to multiple receptors including TLR-4, TLR-2, TLR-9 and RAGE to activate microglia and macrophages. Pharmacological interventions to block HMGB1 have shown a reduction in infarct size [12] and a genetic deletion of its receptor RAGE, similarly, has reduced ischemic brain damage in a mouse model of cerebral ischemia [12]. Interestingly, RAGE expression on infiltrating cells is required to mediate post-stroke inflammation [12] revealing the importance of HMGB1 and RAGE axis to mediate neuronal-glial/macrophage cross talk [36].

#### 2.2.4. Peroxiredoxins

Peroxiredoxins (Prx) are a family of intracellular antioxidant proteins, which upon extracellular release behave as potent DAMPs to upregulate inflammation [37]. In ischemic stroke patients, high levels of Prx-1 and low levels of Prx-5 are reported [38,39]. In a MCAO model of ischemic stroke, peroxiredoxin proteins such as Prx-1, Prx-2, Prx-5 and Prx-6 were found to be released from the injured brain cells and led to the increased expression of proinflammatory cytokines such as IL-23 from macrophages owing to the ligation of TLR-2 and TLR-4 [37].

#### 2.2.5. Cytokines as DAMPs

Both interleukin (IL)-1α and IL-33 belong to IL-1 family of cytokines and are dual-function alarmins [28,40]. IL-1α released from necrotic cells upregulates inflammation and leads to a robust infiltration of neutrophils [41]. Both the precursor and the active form of IL-1α after cleavage of around 100 amino acids from the N-terminus are found to mediate sterile inflammation [42]. IL-1α has been found as the dominant form of IL-1, whose expression is upregulated in microglia as early as 4 h after ischemia-reperfusion insult in areas of focal neuronal damage and penumbra [43]. Similarly, in a subtype of hemorrhagic stroke, in subarachnoid hemorrhage (SAH), IL-1α has also been shown to upregulate the early inflammation and later on, was also associated with a most frequent and feared complication of SAH—the cerebral vasospasm (CVS) [28]. However, contrary to this, intravenous or intra-arterial exogenous administration of IL-1α in subpathological doses offers neuroprotection and neurorestoration in both acute and subacute phases of experimental ischemic stroke [44].

Despite being nominated as a dual-function alarmin like IL-1α, the role of IL-33, its receptor ST2 (suppressor of tumorigenicity 2) and soluble decoy receptor sST2 after ischemic stroke is controversial [28,40,45,46]. IL-33 promotes Th2 response and modulates microglia/macrophages to M2 phenotype [45].

#### 2.2.6. Extracellular-Matrix-Derived DAMPs

Among the expanding list of DAMPs, various matricellular DAMPs such as fibronectin, tenascin C, heparan sulphate, biglycan, versican, and hyaluronan have been described to upregulate inflammation after brain injury [28,46]. Hyaluronan, a component of extracellular matrix that upregulates inflammation via TLR-4 mediated pathway, has been shown to increase in the plasma of acute stroke patients [47,48]. Previously, a study has shown increased levels of serum hyaluronan after ischemic stroke over several days, peaking around day 7, and also in the brain tissues harvested upon autopsy after ischemic stroke [49]. Similarly, hyaluronan expression was upregulated after experimental ischemic stroke [50]. Tang and colleagues have found an inverted U-shaped association between plasma hyaluronan and clinical outcome suggesting lower and higher plasma hyaluronan levels tend to associate with poor clinical outcome [48]. Interestingly, inhibition of hyaluronidase, the enzyme responsible for the liberation of hyaluronan fragments from the extracellular matrix, has been shown to improve the functional outcomes after ischemic stroke [51]. Expression of another extracellular matrix DAMP, tenascin C, was found to upregulate after experimental ischemic stroke, and interestingly, the knockout of tenascin C compromised the exploratory and surveillance activity of the microglia and led to the increased infiltration of peripheral leukocytes, particularly T cells [52]. In a subtype of hemorrhagic stroke, the subarachnoid hemorrhage, tenascin C has been shown to be associated with neuroinflammation, BBB disruption, neuronal apoptosis, cerebral vasospasm and neurological deficits via upregulation of MAPKs and NFκB [53]. A recent investigation showed neuroprotection, reduction in infarct sizes, protection of the BBB, improved motor functions and increased survival upon siRNA-mediated knockdown of tenascin C in experimental ischemic stroke [54]. Interestingly, plasma tenascin C has also been shown to be an independent predictor of futile recanalization after acute ischemic stroke upon endovascular thrombectomy [55].

### 2.3. Brain–Immune Interaction through Immune Signaling Molecules

Signaling molecules such as cytokines and chemokines released from brain cells can stimulate peripheral and central immune cells, and increased expression of adhesion molecules facilitates the infiltration of leukocytes in the damaged brain. IL-1β is a potent proinflammatory cytokine that can induce release of other proinflammatory cytokines such as IL-6 and TNF-α, which subsequently activate peripheral immune cells [56]. TNF-α is acutely and chronically expressed by neurons, astrocytes and microglia after cerebral ischemia, and is implicated in mediating brain injury through several mechanisms such as BBB disruption, activation of leukocytes and glia, enhanced expression of adhesion molecules, thrombogenesis, apoptotic neuronal death, promotion of glutamatergic excitotoxicity through increased expression of AMPA receptors and inhibition of inhibitory GABA receptors [57]. Experimental cerebral ischemia demonstrates expression of TNF-α as early as 1 h after ischemia induction, reaching maximum at 12 h and persisting till day 5 [57]. TNF-α acts in synergism with IL-1β to enhance neuroinflammation and brain damage after ischemic stroke [57]. The detrimental effects of IL-1β include increased expression of cytokines, chemokines, adhesion molecules, activation of matrix metalloproteinases, disruption of BBB, enhanced leukocyte infiltration, platelet activation, decreased neurogenesis and decreased flow of the blood [58]. Along with TNF-α and IL-1β, IL-6 is also upregulated during the acute inflammatory phase after ischemic stroke and accumulating evidence suggests that elevated IL-6 during acute ischemic stroke is associated with poor functional outcomes [59,60]. Experimental studies suggest neuronal expression of IL-6 as early as 3.5 h after cerebral ischemia, peaking at 24 h and persisting till day 7 [60]. Glial cells, infiltrating monocytes and vascular endothelial cells also contribute to IL-6 expression after experimental cerebral ischemia [60].

Damaged cells during cerebral ischemia release a variety of chemokines with homeostatic or inflammatory function. Chemokines including macrophage inflammatory protein-1α (MIP-1α or CCL3), monocyte chemotactic protein-1 (MCP-1 or CCL2) and regulated upon activation, normal T-cell expressed, and secreted (RANTES or CCL5) have shown to be released during different models of stroke [61,62]. Interestingly, CCL5 was deposited in cerebral vasculature and elevated in systemic circulation and associated with recruitment of leukocytes in mouse models of systemic inflammation and stroke [9,13]. Blocking CCL5 led to a decreased leukocyte infiltration in the ischemic brain and conferred neuroprotection [9,13]. During systemic inflammation, the number of circulating leukocytes is dramatically elevated and CCL5 has been suggested to induce MMP-9 release from leukocytes causing BBB disruption [63,64]. Interestingly, a concomitant influenza A virus infection and stroke led to elevated systemic MMP-9 levels that were associated with increased BBB disruption and intracerebral bleeding after thrombolysis [65]. CCL2 is another chemokine that is a potent mediator of macrophage infiltration into the ischemic tissue. CCL2-deficient mice have been shown to have reduced infiltration of inflammatory cells in the ischemic area and decreased infarct size [66,67]. Similarly, CCL3 is associated with accumulation of monocytes in the ischemic area [68] and CXCL8 recruit neutrophils to the site of ischemia via CXCL8 receptor [69,70]. Furthermore, cerebral ischemia leads to upregulated expression of adhesion molecules on the brain endothelial cells that facilitate trans-endothelial migration. Intercellular adhesion molecule-1 (ICAM-1), P-selectin, E-selectin and integrins have been demonstrated to be overexpressed in animal models of cerebral ischemia and interference with these adhesion molecules reduces leukocyte infiltration and tissue injury [71,72]. Evidence suggests an early expression of P-selectin within 15 min and E-selectin after 2 h of ischemia on endothelial cells, which facilitates leukocyte recruitment and activation in the area of ischemic damage [73]. The above-mentioned evidence highlights the possible therapeutic options in modulating the cross talk between injured brain and immune cells through interfering with cytokines, chemokines and adhesion molecules.

### 2.4. Brain–Immune Interaction via Autonomic Nervous System

The rapid nature of signal transduction in the autonomic nervous system (ANS) suggests that both sympathetic and parasympathetic nervous systems are important gateways for the fast communication between the brain and immune system to mediate immune responses [74,75]. The vagus nerve originating from the brain stem modulates the immune response through the inflammatory reflex, which inhibits cytokine release and reduces inflammation associated tissue injury [76]. Several immune cells including macrophages and T cells are functionally innervated by splenic nerve fibers responsive to vagus nerve stimulation [77]. Vagus nerve fibers terminate in celiac ganglia projecting on to the cell bodies of the splenic nerve fibers that innervate the spleen. The splenic fibers from the celiac ganglia are adrenergic in nature and release norepinephrine. However, these splenic fibers innervate T cells in the white pulp region of the spleen, which produce acetylcholine upon splenic fibers stimulation. Hence, acetylcholine producing memory T-cells in spleen play an integral part of inflammatory reflex [76]. Production of acetylcholine from T-cells, inhibits production of cytokines from macrophages through activating nicotinic acetyl choline receptor (nACh) α7 subunit [78]. Ay et al., have demonstrated that vagus nerve stimulation provides neuroprotection in cerebral ischemia through an unknown mechanism [79]. We have previously shown that treatment with GTS-21, a nicotinic acetylcholine receptor (nACh) α7 subunit agonist dampened the systemic inflammatory response and reduced infarct size in a mouse model of systemic inflammation and stroke [13]. On the contrary, around 98% of nerve fibers innervating the spleen are sympathetic in nature and activate smooth muscle cells via α-1 receptors. Moreover, sympathetic activity can influence the function of invariant natural killer T cells in liver, leading to systemic immune suppression via elevated interferon-gamma (IFN-γ) after experimental cerebral ischemia [80]. Interestingly, blocking β-adrenergic receptors with propranolol or depletion of adrenergic nerve fibers in the liver reversed the phenotype of systemic immune suppression and decreased bacterial infections [80]. Inhibition of α-1 adrenergic receptor with prazosin or application of panadrenergic receptor blocker carvedilol reduced the infarct size in experimental ischemia [81]. All these lines of evidence support the notion that inflammatory reflex could be a relevant mean of interaction between CNS and immune system, and could be exploited as therapeutic target to reduce brain damage during cerebral ischemia.

## 3. Impact of Systemic Inflammation and Infiltrated Immune Cells on Ischemic Brain

The brain and immune system communicate through the above-discussed ways and respond to CNS injury. Both the innate and adaptive immune systems have profound influence on the injured brain and deeply affect the clinical outcome via influencing both neurodegeneration in acute phase and regeneration during post-stroke recovery phase.

### 3.1. Impact of Innate Immune Cells on Ischemic Brain

The resident microglial cells are rapidly activated after cerebral ischemia and express activation markers including MHC-II, Iba-1 and CD11b. Activation of microglia is evident before the neuronal demise and precedes the recruitment of peripheral macrophages [82,83]. Soon after the ischemic insult, activated microglia show extensive arborization and exploratory behavior, and later on dearborization and amoeboid transformation [84]. Microglia are associated with the prompt release of proinflammatory cytokines, display early phagocytic and neurotoxic activities [85]. Reactive microglia/macrophages have been detected to peak around four days after experimental cerebral ischemia [86]. However, depletion of microglia after stroke worsens the damage, and evidence suggests microglia later on contribute towards anti-inflammatory effects through inhibition of astrocytes, phagocytosis of dead cells and neutrophils, release of anti-inflammatory and neurotrophic factors [85]. Neuronal interactions with microglia, for instance, loss of CSF1R ligand IL-34 due to the death of neurons, cause microglial depletion, whereas loss of neuronal CX3CL1 and CD200 increases microglial activation [85].

In contrast, the bone-marrow-derived macrophages infiltrate over a period of hours to days and peak at around day 3–7 [83]. Monocyte infiltration was detected as early as within 24 h after cerebral ischemia [86]. Additionally, during the acute phase, the ischemia-reperfusion injury also activates perivascular macrophages and mast cells [87]. Mast cells degranulate releasing histamine, proteases and TNFα, whereas perivascular macrophages release proinflammatory cytokines. Consequently, these stimuli lead to upregulation of adhesion molecules on endothelial cells and contribute to BBB disruption, thus, paving the way for the recruitment of neutrophils, monocytes and lymphocytes [87].

Neutrophils represent the earliest peripheral myeloid cells recruited after cerebral ischemia and can be detected within an hour in microvessels, reaching a peak 1–3 days after ischemic stroke [85,86]. The detrimental effects of neutrophils are owing to the release of granules containing metalloproteinases (MMP9), elastase and cathepsin G, in addition to the production of highly reactive oxygen and nitrogen species. Interestingly, neutrophils also undergo netosis, a unique type of death which results due to the formation of neutrophil extracellular traps (NETs) which are extracellularly displayed nuclear and mitochondrial DNA scaffolds studded with cytotoxic histones and proteases [85]. NETs have been shown to promote thrombosis and impair thrombolysis with tPA [86]. However, tPA promotes neutrophil transmigration, and neutrophilia or higher neutrophil to lymphocyte ratio are associated with a risk of ischemic to hemorrhagic transformation after tPA administration [86]. Neutrophils showing alternative activation and presenting with upregulated expression of Arg1 and YM1, displaying anti-inflammatory and repair activities, reduced oxidative burst and netosis, need further investigation to delineate the possible neuroprotective effects [85,86].

Both resident microglia and macrophages clear the debris of infarcted tissue [88]. Depending on the nature of the environment, microglia and macrophages can polarize to the classical/inflammatory or the alternate/anti-inflammatory cells. A growing body of evidence shows maintaining the microglia in M2-phenotype (anti-inflammatory microglia) protects neurons against ischemia. We have already demonstrated that a shift of balance of monocytes/macrophages towards alternate/M2- phenotype showed a robust neuroprotection in a mouse model of cerebral ischemia [15]. However, neutrophils infiltrate rapidly within few hours after stroke and peak at day 1–3 [89]. Adhesion molecules such as ICAM-1 and P-selectin facilitate the neutrophil infiltration [90,91]. Infiltrating neutrophils release NO and MMP-9, which play a key role in disrupting BBB. Elevated systemic MMP-9 during concomitant stroke and systemic inflammation correlates with thrombolysis-associated intracerebral bleeding [65]. Furthermore, depletion of neutrophils with an antibody-based approach reduced the infarct size and brain edema in an experimental cerebral ischemia [92]. Dendritic cells from the systemic circulation also infiltrate early at day 1 and reach to a peak at day 3–7, and being antigen-presenting cells modulate the adaptive immune response [93]. Based on above findings, the suppression of neutrophils and polarization of microglia and macrophages towards M2 type might be possible therapeutic options to achieve better outcomes after stroke.

### 3.2. Impact of Adaptive Immunity (Immune Cells) on Ischemic Brain

The adaptive immune system is activated by antigens that are released from the ischemic brain. Lymphocytes start accumulating in ischemic brain within 24 h after stroke [94] and play a critical role in defining the clinical outcome, especially at a later stage. Rag 1 knockout mice lacking mature lymphocytes showed smaller infarct size and better clinical outcome after experimental stroke [95] and implantation of CD3+ T lymphocytes into Rag 1 knockout mice abolished this protective effect [95]. Interestingly, mice deficient in either CD4+ T helper cells or CD8+ T cells exhibited significantly smaller infarct sizes [96] and a selective depletion of either CD4+ or CD8+ cells using antibodies, remarkably reduced the infarct volume in a model of permanent MCAO [97], demonstrating the detrimental role of both subpopulations of T cells. On the other hand, regulatory T cells (Tregs), a subpopulation of CD4+ T helper cells account for only 10% of circulating CD4+ T helper cells, mediated neuroprotection and improved neurological outcome via production of anti-inflammatory cytokines [11]. Recent investigations suggest that Tregs with brain specific signatures were recruited during the chronic phase of stroke and protected against astrogliosis, suppressed neurotoxic astrocytes, and promoted functional recovery [98]. These evidences suggest that a therapeutic approach targeting a selectively specific subpopulation of T cells may provide options to improve post-stroke outcomes.

## 4. Brain–Immune Interaction after Cerebral Ischemia and Therapeutic Options

Post-stroke neuronal death is a progressive process and continues over days to weeks after the initial insult [99]. Keeping in view the brain–immune interaction, there are multiple options that could be of therapeutic interest.

### 4.1. Targeting DAMPs and Their Receptors

Necrotic cells release highly active proinflammatory danger signal molecules that enhance the neuroinflammation through receptors like RAGE and TLRs by activating multiple downstream pathways in the neural cells [7,12]. Blocking the release of danger signal molecules or strategies to neutralize them with antibodies or interference with their receptors to block the cellular effects could be a possible therapeutic implication.

### 4.2. Targeting Immune-Signaling Molecules

Several immune-signaling molecules, such as cytokines, chemokines and adhesion molecules, play an important role in orchestrating the inflammatory response after cerebral ischemia. For instance, TNF-α antagonism through soluble TNF receptor (sTNFR) or monoclonal antibodies directed against TNF-α provide neuroprotective effects after cerebral ischemia [57]. Etanercept, an inhibitor of TNF-α, shows neuroprotection after experimental cerebral ischemia and also improves clinical outcomes in the context of chronic stroke [100,101]. As mentioned above, TNF-α enhances the detrimental effects of IL-1β, and antagonism of IL-1β either through anti-IL-1β antibodies or administration of the IL-1Receptor antagonist (IL-1RA) has been shown to be neuroprotective and improves outcomes after ischemic stroke [58]. Elevation of IL-6 levels also accompany TNF-α and IL-1β levels after ischemic stroke; however, antagonism of IL-6 has yielded conflicting results in the experimental setting. Administration of IL-6 receptor antagonist (IL-6RA) monoclonal antibody in a mouse model of ischemic stroke was found to increase the infarct volume, peri-infarct apoptosis and motor dysfunction [102]. However, recent studies have shown neuroprotective effects of an FDA-approved IL-6RA, tocilizumab, after experimental cerebral ischemia [103,104]. In one study, rats underwent cerebral ischemia after 7 days of treatment with tocilizumab, and tocilizumab administration prevented apoptosis [103]. Administration of tocilizumab 4 h after cerebral ischemia induction was found to protect against acute and long-term larger brain infarcts and brain atrophy in aged male mice [104].Various experimental and clinical studies have also targeted adhesion molecules to ameliorate the effects of infiltrating immune cells in the CNS after cerebral ischemia. Antibody-mediated antagonism of P-selectin and E-selectin was found to be neuroprotective after experimental cerebral ischemia [105,106]. However, a clinical phase II study investigating the mucosal tolerance and shift of the Th1 response to Th2 upon repeated intranasal spray of E-selectin to prevent recurrent strokes was terminated without disclosing any findings [107]. In a study targeting endothelial integrin ligand ICAM-1, anti-ICAM-1 murine antibody was shown to be effective in experimental cerebral ischemia, but offered no neuroprotective benefits in the clinical phase III trial, rather was associated with adverse clinical outcomes [108]. Several integrins such as LFA-1 (CD11a CD18, αLβ2), Mac-1 (CD11b CD18, αMβ2), VLA-4 (CD49d/CD29, α4β1), and VLA-5 (CD49e/CD29, α5β1) have been shown to be upregulated after cerebral ischemia and their inhibition has been shown to confer neuroprotective effects after experimental cerebral ischemia [73]. However, fruitful clinical studies for the treatment of ischemic stroke that are based on targeting integrins are still underdevelopment [73].

### 4.3. Targeting the Autonomic Nervous System

Experimental data suggest that the vagus nerve controls the release of multiple cytokines from peripheral macrophages through inflammatory reflex. Stimulation of nicotinic acetyl choline receptor (nACh) α7 subunit is a key in the vagus control of immunity. Vagus nerve stimulation can protect the brain from ischemia and in line with these findings, blocking the activity of sympathetic nervous system can protect against the post-stroke immune depression and infection-mediated deaths [79,80]. Besides vagus nerve stimulation, selectively targeting nicotinic acetyl choline receptor (nACh) α7 subunit or adrenergic receptors could also be a therapeutic implication for the treatment of stroke.

### 4.4. Targeting Polarization of Microglia/Macrophages towards M2 Type Phenotype

The polarization towards a specific population depends on the microenvironment. M2 type microglia can attenuate activation of invading inflammatory cells via release of IL-10 and TGF-β [11,109]. Interestingly, monocytes/macrophages express molecules that enhance angiogenesis such as VEGF, TGF-β and MMP-9 [110,111] providing post-stroke recovery. Moreover, monocytes and microglia release neural trophic factors such as BDNF, CCL2 and SDF-1 to enhance neurogenesis and facilitate migration of newborn neurons [112,113,114]. Interestingly, CD14+ monocytes have themselves the capacity to differentiate into neuronal lineage [115]. Furthermore, in the process of remyelination, oligodendrocyte precursor cells (OPC) are necessary. M1 type microglia and macrophages cause oligodendrocyte precursor cell (OPC) death, whereas M2 type cells enhance chemotaxis and differentiation of OPC cells [116,117]. IL-4 and IL-33 administration was found to polarize towards M2 microglia/macrophages and confers neuroprotection after cerebral ischemia [45,118]. Besides targeting molecules released by necrotic tissue, polarization of microglia and macrophages towards a beneficial subtype could be an interesting strategy to treat stroke.

### 4.5. Targeting Polarization of Specific T Cell Response towards Th2 and Regulatory T-Cells

Depending upon microenvironment and cellular interactions with innate immune cells, T helper cells can polarize towards Th1, Th2, Th17 or towards regulatory T cells. The polarization of T cells can critically influence progression of injury, angiogenesis and tissue repair. On one hand, the polarization of T helper cells towards Th1 type CD4+ cells inhibit angiogenesis through the release of IFN-γ [119] and on the other hand, Th2 type CD4+ cells and regulatory T cells promote angiogenesis via releasing VEGF and TGF-β [120,121]. Moreover, Th1 type cells have the potential to induce demyelination and Th2 type cells improve OPCs [116] to enhance remyelination and regeneration. Hence, either a selective polarization of T cells towards Th2 type and T regulatory cells or adoptive transfer of these cells could be an important therapeutic implication.

### 4.6. Evidence of Stem Cell Signals to Instruct Immune Micro Milieu

Neurogenesis is complex process where new cells are born from stem cells in the brain. Despite the ongoing debate regarding the biological relevance of adult neurogenesis [122], accumulating evidence suggests misregulated neurogenesis is promoting aging-related brain diseases [123]. More recently, mostly emerging from discoveries in cancer research, the fatal tango pair of stem cells and immunity [124] is brought into attention as how stem cell signals may fundamentally contribute to disease course and therapy resistance [125,126,127]. Some most recent observations indicate the importance of stem cell factors in immune regulation during stroke [128,129,130] and brain diseases including non-cancer problems [131,132,133,134].

Many different types of stem-cell-based therapies consisting of neural stem cells, mesenchymal stem cells, embryonic stem cells, and human induced pluripotent stem-cell-derived neural stem cells have been evaluated as a potential therapy against ischemic stroke both in preclinical and clinical settings [135]. However, the time frame windows for implementation of these stem cell based therapies and the mechanism through which stem cells exert their beneficial effects still require further investigations. Modulation of the immune milieu by these stem cells may be one of the underlying mechanisms. Interestingly, in vitro co-culture of macrophages/microglia with mesenchymal-derived stem cells (MSCs) have shown to promote M2 microglia/macrophage polarization [128]. Similarly, in vivo transplantation of MSCs after experimental MCAO in rodents have shown decreased infarct volumes and increased functional recovery through polarization of macrophages/microglia towards the neuroprotective and neuroregenerative M2 phenotype [128]. Intriguingly, alternatively activated microglia serve as the source of IGF-1 (insulin like growth factor-1) in the subventricular zone (SVZ) and promote proliferation, differentiation and migration into the striatum after experimental ischemic stroke [136]. Similarly, anti-inflammatory Tregs were shown to promote neural stem cells proliferation in the SVZ after experimental cerebral ischemia through IL-10 [137]. An experimental cerebral ischemia study involving administration of bone marrow mononuclear cells administration showed that the proliferation of neural progenitor/stem cells was dependent on the proliferation of endothelial cells [138]. Further, investigations seeking efficacy, time frame windows, stem-cell-based therapy alone or in combination with thrombolytic or endovascular thrombectomy, and the potential mechanisms of stem-cell-based therapies are urgently required [135,139]. Our limited knowledge on stem cell signal driven pathology presents a lack of literature evidence.

## 5. Discussion

**Figure 1:**
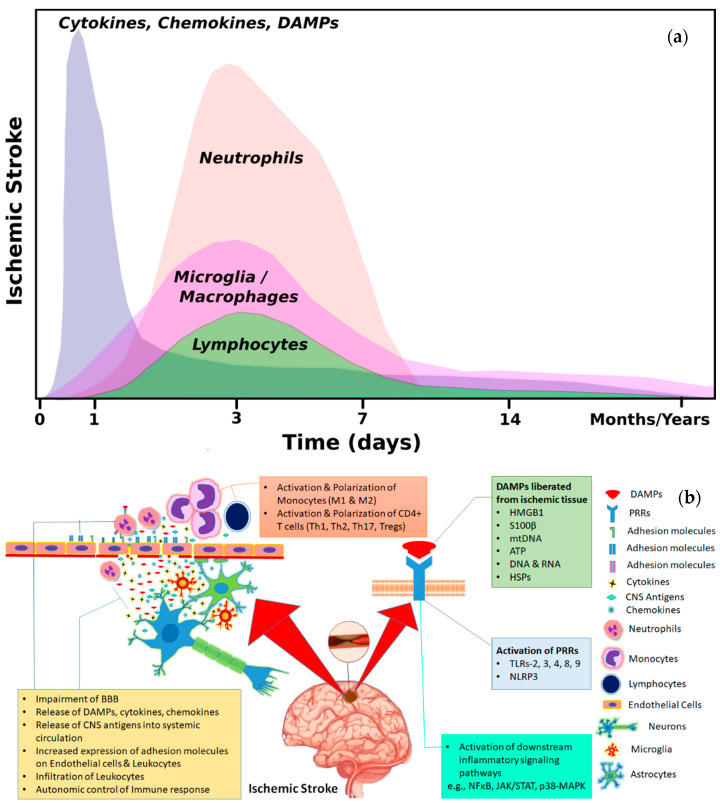
(**a**). Temporal dynamics of major immune cell types and inflammatory mediators after ischemic stroke. The release of DAMPs from the ischemia injured cerebral tissue leads to the activation of microglia/macrophages which release chemokines and cytokines leading to the infiltration of peripheral immune cells. Neutrophils and monocytes/macrophages are recruited along with lymphocytes from the periphery to the ischemic cerebral tissue. (**b**). A schematic representation of brain–immune interactions taking place after cerebral ischemia. Brain-derived antigens, DAMPs, cytokines and chemokines are released into systemic circulation through the impaired blood–brain barrier (BBB) after cerebral ischemia and lead to activation and polarization of immune cells (M1/M2, Th1/Th2, Th17/Tregs). Increased expression of adhesion molecules on endothelial cells and immune cells lead to increased infiltration by the leukocytes in the already damaged ischemic brain causing secondary damage. DAMPs: damage-associated molecular patterns; PRRs: pattern recognition receptors; TLRs: Toll like receptors; HSPs: heat shock proteins; M1: classically activated proinflammatory monocytes/macrophages; M2: alternatively activated anti-inflammatory monocytes/macrophages; Tregs: T regulatory cells; Th cells: CD4+ T helper cells.

Stroke remains the major contributor to worldwide mortality and morbidity despite enormous advancements in biomedical sciences and the availability of limited clinical approaches to treat it. State of the art research suggests a critical role of inflammation after stroke that determines the clinical outcome [140]. Discerning various endogenous mechanisms that limit or neutralize excessive proinflammatory responses could lead to novel therapeutic options for stroke. Additionally, several risk factors e.g., inheriting various class I HLA molecules and killer immunoglobulin like receptors (KIRs) alleles, which play important role in T cell and NK cell responses, may determine the protective or detrimental impact after ischemic stroke [141]. All the evidences presented above highlight an importance of various axes of brain–immune interactions in mediating the disease and contributing to stroke outcomes. Modulation of these interactions in a correct way could serve as therapeutic interventions. Figure 1a summarizes the time course of major immune cell types after cerebral ischemia and Figure 1b shows the major molecular and cellular targets that may be targeted to modulate the brain–immune interactions after stroke.

Various DAMPs can be released after ischemic insult into the periphery and may activate the peripheral immune system. Owing to compromised BBB, several antigens specific to CNS can be unveiled for the development of autoreactive adaptive immunity. Several signaling molecules such as cytokines, chemokines and adhesion molecules may also play an important role in immune activation and inflammation. The innate immune cells such as microglia/macrophages can polarize to neurodamaging (M1) and neuroprotective (M2) phenotypes as do the adaptive CD4+ T helper cells to Th1, Th2 and Th17 cells, respectively. Intriguingly, discovery of the autonomic reflex control of immunity provides another prompt mechanism of regulation of the inflammatory cascades. So, ample evidence highlights the critical roles of these brain–immune interactions, which can be modulated to develop future promising therapeutic entities.

Among various DAMPs, HMGB1 represents a prototypical protein transcription factor that has been increasingly investigated for its role in post-ischemic inflammation and its modulation as a therapeutic target. For instance, antibody-mediated neutralization of HMGB1 or its antagonism via HMGB1 box A or glycyrrhizin can be neuroprotective in ischemic stroke models [12,142]. Interestingly, ameliorating the HMGB1-RAGE interaction via administering a decoy soluble RAGE receptor was found to be neuroprotective in ischemic stroke model [12]. Similarly, peroxiredoxins were released after 12 h of stroke onset and antibody mediated neutralization of peroxiredoxins downregulated the inflammation and prevented ischemic brain damage [37]. Interestingly, upregulation of the scavenger receptors such as MSR1 on infiltrating myeloid cells by modulating the Mafb expression through administration of retinoic acid receptor (RAR) agonist Am80 led to the enhanced clearance of various DAMPs (HMGB1, peroxiredoxins, S100A8, S100A9) from the ischemic brain and prevented delayed inflammation and damage [143].

Microglial/macrophage activation inhibition via administration of minocycline was found to be neuroprotective in two phase two clinical studies [144,145]. Macrophages/microglia may be polarized to neuroprotective, alternatively activated (M2) phenotype either by the use of miRNAs such as miRNA-181a or exploiting the epigenetic control of macrophage polarization to achieve better outcomes in stroke. Alternatively activated (M2) microglia/macrophage polarization has also been shown in experimental stroke models with drugs such as minocycline, azithromycin, exendin-4, metformin and rosiglitazone [146,147]. Several traditional and Chinese alternative medicine ingredients have shown the potential to promote alternatively activated (M2) response and neuroprotection in experimental ischemic stroke settings e.g., ginkgolide B, malibatol A, hyperforin, thiamet G and noggin [147]. Non-invasive vagus nerve stimulation may also be employed to polarize macrophages to alternatively activated (M2) type that affords neuroprotection against ischemia-reperfusion injury [148]. Several other agents discussed above can modify the autonomic reflex and provide neuroprotective effects in ischemic brain injury.

Depletion of T cells from the ischemia afflicted brain represents another potential therapeutic intervention [149]. Among different T helper cell subsets, promotion of Th1 cell response (proinflammatory) has been shown to increase the infarct volumes and its suppression with a promotion of Th2 or Treg responses (anti-inflammatory) offered neuroprotection in ischemic stroke models [11,150,151,152,153]. A few agents such as glatiramer acetate or statins as well as aluminum hydroxide (used as vaccine adjuvant) have been shown to induce Th2 response leading to neuroprotection and regeneration [150].

Several proinflammatory mediators released after ischemic brain insult represent attractive targets of intervention. For instance, the effects of IL-1β released after stroke can be neutralized by administration of IL-1β receptor antagonist. Preclinical results and a clinical phase II study have demonstrated beneficial and safety potential of IL-1β receptor antagonist after ischemic stroke [58,154]. Over the past years, an array of neutralizing antibodies have been developed that target a wide range of proinflammatory mediators including TNF-α, IL-12, IL-23, IL-17, and are under investigation in clinical trials for various neurological and other diseases [155,156,157,158]. Anti-adhesion molecules antibodies may also be employed to curb the inflammatory response such as Natalizumab, anti-α4 integrin, have been found to be effective in the setting of multiple sclerosis [140]. However, the clinical development of effective anti-adhesion molecules against ischemic stroke is still awaited [73]. Accumulating evidences support an important role of inflammation that is upregulated via various brain–immune interactions as discussed above and represents important therapeutic targets of intervention to achieve better outcomes after stroke.

Given the importance of stem cells in the brain for various brain functions, stimulating or modulating the activity of those pathways in the context of the disease, is an attractive opportunity to sustainably intervene in the neuro-immune axis. Although, the field is far from a mutual consensus on how stem cell, or stem cell signals, instruct neuroimmunity, the authors believe further research in this segment shall lead to innovative new traits to treat or improve therapy surveillance of cerebral ischemia. The inclusion of stem cell investigation in inflammation-directed projects on cerebral pathologies should be promoted.

## 6. Conclusions

Recent advancements in experimental research have unveiled the interactions taking place between the injured brain and immune system after ischemic stroke. These interactions represent potential modulatable targets of therapeutic intervention using pharmacological agents with an ultimate goal to improve the quality of life of stroke-afflicted patients and reduce mortality.

## Data Availability

Not applicable.
